# Deep regression analysis for enhanced thermal control in photovoltaic energy systems

**DOI:** 10.1038/s41598-024-81101-x

**Published:** 2024-12-23

**Authors:** Wael M. Elmessery, Abadeer Habib, Mahmoud Y. Shams, Tarek Abd El-Hafeez, Tamer M. El-Messery, Salah Elsayed, Ahmed E. M. Fodah, Taha A. M. Abdelwahab, Khaled A. M. Ali, Yasser K. O. T. Osman, Mohamed F. Abdelshafie, Gomaa G. Abd El-wahhab, Abdallah E. Elwakeel

**Affiliations:** 1 Agricultural Engineering Department, Faculty of Agriculture, Kafrelsheikh University, Kafr El-Shaikh, 33516 Egypt; 2 International Research Centre “Biotechnologies of the Third Millennium”, Faculty of Biotechnologies (BioTech), ITMO University, St. Petersburg, 191002 Russia; 3 Department of Machine Learning and Information Retrieval, Faculty of Artificial Intelligence, Kafrelsheikh University, Kafr Elsheikh, 33516 Egypt; 4 Department of Computer Science, Faculty of Science, Minia University, Minia, 61519 Egypt; 5 Computer Science Unit, Deraya University, Minia University, Minia, 61765 Egypt; 6 Agricultural Engineering, Evaluation of Natural Resources Department, Environmental Studies and Research Institute, University of Sadat City, Cairo, 32897 Minufia Egypt; 7 College of Agricultural Engineering, Al-Azhar University, Cairo, 11768 Egypt; 8 College of Engineering, South China Agriculture University, Guangzhou, China; 9 Agricultural Engineering Department, Faculty of Agriculture and Natural Resources, Aswan University, Aswan, 81528 Egypt

**Keywords:** Photovoltaic solar panels, Cooling systems monitoring, Thermal imaging, Deep learning, Image segmentation, Computer vision, Performance optimization, Computer science, Information technology, Scientific data, Energy science and technology, Energy harvesting, Energy infrastructure, Energy storage, Renewable energy, Thermoelectric devices and materials

## Abstract

Efficient cooling systems are critical for maximizing the electrical efficiency of Photovoltaic (PV) solar panels. However, conventional temperature probes often fail to capture the spatial variability in thermal patterns across panels, impeding accurate assessment of cooling system performance. Existing methods for quantifying cooling efficiency lack precision, hindering the optimization of PV system maintenance and renewable energy output. This research introduces a novel approach utilizing deep learning techniques to address these limitations. A U-Net architecture is employed to segment solar panels from background elements in thermal imaging videos, facilitating a comprehensive analysis of cooling system efficiency. Two predictive models—a 3-layer Feedforward Neural Network (FNN) and a proposed Convolutional Neural Network (CNN)—are developed and compared for estimating cooling percentages from individual images. The study aims to enhance the precision and reliability of heat mapping capabilities for non-invasive, vision-based monitoring of photovoltaic cooling dynamics. By leveraging deep regression techniques, the proposed CNN model demonstrates superior predictive capability compared to traditional methods, enabling accurate estimation of cooling efficiencies across diverse scenarios. Experimental evaluation illustrates the supremacy of the CNN model in predictive capability, yielding a mean square error (MSE) of just 0.001171821, as opposed to the FNN’s MSE of 0.016. Furthermore, the CNN demonstrates remarkable improvements in mean absolute error (MAE) and R-square, registering values of 1.2% and 0.95, respectively, whereas the FNN posts comparatively inferior numbers of 3.5% and 0.85. This research introduces labeled thermal imaging datasets and tailored deep learning architectures, accelerating advancements in renewable energy technology solutions. Moreover, the study provides insights into the practical implementation and cost-effectiveness of the proposed cooling efficiency monitoring system, highlighting hardware requirements, integration with existing infrastructure, and sensitivity analysis. The economic viability and scalability of the system are assessed through comprehensive cost-benefit analysis and scalability assessment, demonstrating significant potential for cost savings and revenue increases in large-scale PV installations. Furthermore, strategies for addressing limitations, enhancing predictive accuracy, and scaling to larger datasets are discussed, laying the groundwork for future research and industry collaboration in the field of photovoltaic thermal management optimization.

## Introduction

The ever-growing demand for electrical power, driven by industrialization and technological advancements, has intensified the focus on optimizing the efficiency of power generation systems.

Photovoltaic (PV) solar power has emerged as a critical renewable energy source, but maintaining high electrical efficiency relies heavily on effective panel cooling systems^1^. Various cooling systems are used in photovoltaic (PV) systems to improve energy conversion efficiency and prevent performance loss. Passive and active cooling methods are applied on the front and back surfaces of PVs under different working conditions. These cooling techniques include heat recovery units, water-cooled heat sinks, and backside water cooling systems^2–4^. It is important to note that many of these cooling methods, particularly those involving active cooling with fluids, are characteristic of photovoltaic/thermal (PV/T) systems. PV/T systems are hybrid solar systems that convert solar radiation into both electricity and useful thermal energy simultaneously. These systems not only cool the PV panels to maintain electrical efficiency but also harness the thermal energy for applications such as water heating or space heating, thereby increasing the overall energy conversion efficiency of the system. Therefore, Monitoring and optimizing cooling dynamics for solar plant performance and longevity is crucial, especially considering the limitations of traditional temperature probes in characterizing surface variability across panels. Novak et al.^5^ proposed an intelligent automation system that monitors parameters such as cooling medium amount, temperature, flow control, panel temperature, and local weather conditions to evaluate the conditions for active smart cooling. Singh and Yadav^6^ developed an experimental setup for solar panel cooling using water cooling arrangements and optimized the input parameters to achieve improved efficiency and module temperature. Herfatmanesh et al.^7^ conducted experiments to explore the effects of solar PV surface temperature on output performance and proposed a cooling system that increased efficiency by close to 50%. Gujar et al.^8^ designed a PV-panel cooling system using fins and phase change material, which maintained the temperature below 50 °C and increased conversion efficiency by 5–7%. Liu et al.^9^ summarized various solar cell cooling technologies, including traditional cooling methods and new technologies like heat pipe cooling and microchannel cooling.

Thermographic Non-Destructive Test (TNDT) is a common method for diagnosing faults in PV systems. It uses infrared thermography to examine the operating conditions of the system. TNDT analysis allows for the measurement of cell temperature and the detection of localized overheating in the module, known as hotspots, by capturing thermographic images that show the surface temperature of the module in each pixel^10^. This technique enables the identification of areas of potential defects without directly accessing the module. By detecting hotspots, TNDT can help prevent performance loss and ensure the safety of the PV system^11,12^.

Thermal imaging has been widely proposed as a solution for non-invasive, high-resolution visualization of temperature patterns. Yet analytical approaches for extracting actionable insights from thermal video remain limited^13^.

Recent literature reflects a growing interest in utilizing deep learning techniques, such as convolutional neural networks (CNNs), for analyzing thermal imaging in the context of predictive insights and condition monitoring. These techniques have been applied to various fields, including nuclear power plants^14^, crack detection in inductive thermography^15^, classification of patterns in thermographic images of a bench grinder^16^, early pavement damage detection^17^, and defect inspection in artwork using principal component analysis^9^. These studies demonstrate the effectiveness of deep learning in extracting features and enhancing the quality of thermal images, leading to improved detection and classification of defects and faults. CNNs have shown promise in detecting and classifying faults in photovoltaic (PV) systems using thermal images^10,18^. Deep learning methods, such as variational autoencoders (VAEs), have been used to expand the data set and improve the accuracy of fault classification^19^. Another approach is to use a deep-learning-based defect detection method that addresses the challenges of limited data and data imbalance, achieving high accuracy in identifying the presence or absence of defects and different defect types^20^. Additionally, a high-precision algorithm has been proposed for detecting and classifying defects in PV panels, achieving a detection accuracy of 92.0%^21^. Furthermore, thermal CNNs have been used for the detection, quantification, and on-field localization of overheated regions on PV arrays, with the best performance achieved by the FPN-DenseNet121 model. However, there is limited research on using deep learning on thermal video to directly quantify and forecast cooling efficiency for optimizing PV performance.

Data-driven regression models based on CNNs represent a highly promising approach for precisely estimating cooling effectiveness from thermal imagery^22^. Recent applications of regression CNNs in related domains have shown strong capabilities for predicting system parameters from spatial temperature maps^23^. However, tailored datasets and models are needed to focus specifically on correlating thermal patterns with PV cooling efficiency. The development of automated pipelines for generating labeled cooling efficiency data from thermal plant videos could enable robust CNN regressors^24^. The integration of deep CNN architectures for regression with extensive annotated thermal datasets has the potential to significantly advance non-invasive quantification and monitoring of photovoltaic cooling dynamics. This will provide crucial insights for predictive maintenance and efficiency improvements in solar power systems^25^.

### Problem statement

Despite advancements in thermal management for photovoltaic (PV) solar panels, existing methods for quantifying cooling efficiency often lack the precision necessary for optimizing PV system maintenance and energy output. Traditional temperature probes fail to capture spatial variability in thermal patterns, which is crucial for accurate cooling system assessment.

### Research question

How can deep learning techniques be applied to thermal imaging data to improve the quantification of cooling system efficiency in PV solar panels, and what impact does this have on the optimization of renewable energy output?

### Practical applications

Our deep regression analysis method goes beyond simply obtaining temperature information. It provides a real-time, non-invasive way to quantify cooling system efficiency across large-scale photovoltaic installations. This capability has several practical applications:

- Predictive Maintenance: By continuously monitoring cooling efficiency, operators can identify underperforming or failing cooling systems before they significantly impact PV output. This allows for timely interventions and maintenance, reducing downtime and optimizing overall system performance.
- Optimization of Cooling Strategies: For PV systems with active cooling capabilities (such as PV/T systems, as you correctly point out), our method provides valuable feedback on the effectiveness of different cooling strategies. This data can be used to fine-tune cooling parameters, such as flow rates or activation thresholds, to maximize efficiency and minimize water/energy consumption.
- Design Improvements: The spatial resolution of our thermal analysis can highlight areas of panels that consistently experience higher temperatures. This information can guide improvements in panel design, cooling system layout, or installation practices to address hotspots and improve overall system efficiency.

### Improving cooling system performance

Our method contributes to improved cooling system performance in the following ways:

- For PV/T Systems: In photovoltaic-thermal hybrid systems, our method provides precise, real-time feedback on cooling efficiency. This data can be integrated into control systems to dynamically adjust coolant flow rates, optimizing the balance between electrical output and thermal energy harvesting.
- For Passive Cooling Systems: While passive cooling systems cannot actively reduce temperatures, our method helps in assessing the effectiveness of various passive cooling techniques (e.g., enhanced convection, reflective coatings). This information is valuable for comparing different passive cooling strategies and guiding future installations.
- Performance Verification: Our method offers a way to verify the performance of newly installed or upgraded cooling systems, ensuring they meet design specifications and operate efficiently under real-world conditions.

#### Indirect improvement of electricity production

While our system doesn’t directly cool PV modules, the insights it provides can lead to improved electricity production through:

- Informed Decision Making: By providing accurate, real-time data on cooling efficiency, our system enables operators to make informed decisions about when to clean panels, adjust tilt angles, or implement other temperature management strategies.
- Long-term Performance Optimization: The data collected over time can inform better system designs, cooling strategies, and maintenance schedules, all of which contribute to improved long-term electricity production.
- Integration with Smart Grid Systems: In large-scale installations, our cooling efficiency data can be integrated with smart grid management systems to optimize overall energy production and distribution based on real-time panel performance.

## The literature review

Harnessing solar energy has gained popularity as an efficient method to power homes, businesses, and other utilities. One such efficient method is through the use of solar thermoelectric generators, which transform thermal energy into electricity, offering a wide range of applications, such as heating water and powering electronics.

Li et al.^26^ emphasize the need for complex control and monitoring systems to maintain efficiency in thermal energy harvesting systems, which can be costly to implement and maintain. Ma et al.^27^ illustrated the potential of solar energy as a renewable, clean, and abundant source of energy. However, one of the key challenges is achieving maximum energy harvesting. Trappey et al.^28^ presented solar thermoelectric absorbers as a method to harness solar energy and convert it into electricity. However, several issues need addressing to maximize energy harvesting with solar thermoelectric absorbers. Sun et al.^29^ discussed the considerable environmental impact of thermal energy harvesting. Some thermal energy harvesting systems rely on fossil fuels to generate heat, leading to an increase in air pollution. Moreover, these systems may require large amounts of water for cooling, resulting in significant water wastage. Sajedian et al.^30^ emphasized the importance of thermal energy harvesting in reducing emissions and improving energy efficiency. However, several issues need to be addressed to make this technology a feasible solution for many applications. These issues include overall efficiency, cost, size and location requirements, and environmental impacts. Ahmad et al.^31^ discussed that with the right solutions, these issues can be addressed, making thermal energy harvesting a more practical option for a wide range of applications.

Zhang, X et al.^32^ addressed the high costs involved in thermal energy harvesting systems, which can often be a deterrent for many applications. Another problem related to thermal energy harvesting is the large area required to capture and store energy, which can significantly influence the project’s feasibility and cost depending on its size and location. The availability of heat sources in some regions can also pose challenges, as they might lack a reliable heat source. Lin et al.^33^ expressed one such challenge is the potential for low efficiency. The efficiency of thermal energy harvesting systems depends on the temperature difference between the waste heat source and the ambient environment, as well as the conversion system’s efficiency. Gorjian et al.^34^ discussed the cost of the absorber as a significant factor in maximizing energy harvesting. Solar thermoelectric absorbers are generally more expensive than other solar energy harvesting technologies, such as photovoltaics. Therefore, finding ways to reduce the absorber’s cost is crucial to maximize energy harvesting.

Bai et al.^35^ spoke of the growing field of thermal energy harvesting technology and its potential to drastically change our energy consumption and production practices. This involves capturing and using waste heat produced by industrial processes, vehicles, and other energy sources. Despite its potential, thermal energy harvesting faces many significant challenges Gao et al.^36^ discussed another challenge being the system’s ability to withstand the harsh conditions of industrial processes and exhaust systems. High temperatures, corrosive chemicals, and other extreme conditions can quickly degrade these systems’ components, leading to premature failure, and resulting in financial losses and wasted energy. Varga et al.^37^ explained that thermal energy harvesting is a promising technology field with the potential to revolutionize energy production and consumption. With adequate investments, research, and development, these systems can overcome the challenges and become a crucial part of our energy infrastructure.

Elsheikh et al.^38^ elaborated on the process of thermal energy harvesting, which involves the extraction of energy from heat sources in the environment and converting it into practical energy. The significance of thermal energy harvesting has been growing in recent years, as it helps in reducing emissions, enhancing energy efficiency, and ensuring a reliable energy supply. There are, however, critical issues that need resolving to make thermal energy harvesting a feasible solution for numerous applications. Liu et al.^39^ highlighted the efficiency problems associated with thermal energy harvesting. Heat energy is challenging to capture and store, and a considerable amount of energy is lost during the conversion process, which results in a lower overall efficiency of thermal energy harvesting systems compared to other energy production forms. Table 1 presents a thorough examination of related research efforts focusing on thermal energy harvesting.

**Table 1:** Overview of challenges and metrics in thermal energy harvesting.

Author	Classification	Identified issues	Metrics
Li et al.^26^	Implementation complexity	Complexity and cost associated with implementing control and monitoring systems.	Highlights the challenges related to implementing and maintaining control systems for thermal energy harvesting systems.
Ma et al.^27^	Energy maximization	Focuses on challenges in maximizing energy harvesting from solar sources.	Emphasizes the importance of maximizing energy harvesting from solar sources for sustainable energy production.
Sun et al.^29^	Environmental impact	Environmental impacts associated with thermal energy harvesting, particularly air pollution from fossil fuel combustion.	Raises concerns about the environmental consequences of certain thermal energy harvesting methods reliant on fossil fuel combustion.
Trappey et al.^28^	Solar thermoelectric absorbers	Issues in optimizing energy harvesting with solar thermoelectric absorbers.	Discusses challenges and opportunities in optimizing energy harvesting using solar thermoelectric absorbers.
Ahmad et al.^31^	Transformative potential	The transformative potential of thermal energy harvesting in revolutionizing energy consumption and production.	Highlights the role of thermal energy harvesting in utilizing waste heat from industrial processes and vehicles to improve energy sustainability.
Zhang et al.^32^	Spatial requirements	The spatial requirements and feasibility of thermal energy harvesting projects.	Highlights the importance of project size and location in determining the feasibility and cost-effectiveness of thermal energy projects.
Sajedian et al.^30^	Comprehensive challenge overview	A comprehensive overview of challenges including efficiency, cost, spatial requirements, and environmental impacts.	Suggests that addressing these challenges is crucial for enhancing the viability of thermal energy harvesting across various applications.
Lin et al.^33^	Temperature differentials	Limitations in temperature differentials impacting system efficiency.	Notes the importance of temperature differentials in optimizing the efficiency of thermal energy harvesting systems.
Gorjian et al.^34^	Cost considerations	Cost considerations associated with solar thermoelectric absorbers.	Discusses cost implications and challenges in the adoption of solar thermoelectric absorbers for energy harvesting.
Bai et al.^35^	Efficiency issues	The primary challenge of low efficiency in thermal energy harvesting systems.	Stresses the significance of addressing low-efficiency issues to maximize the effectiveness of thermal energy harvesting technologies.
Liu et al.^39^	Efficiency comparison	Challenges related to the efficiency of thermal energy harvesting systems and their comparison with other energy production methods.	Emphasizes the lower efficiency of thermal energy harvesting systems compared to alternative energy production methods.
Elsheikh et al.^38^	Emission reduction and energy efficiency	The importance of thermal energy harvesting in reducing emissions, enhancing energy efficiency, and ensuring reliable energy supply.	Advocates for thermal energy harvesting as a critical solution for addressing environmental concerns and enhancing energy reliability.
Gao et al.^36^	Durability and harsh conditions	Concerns regarding system durability and performance under harsh conditions.	Emphasizes the need for thermal energy harvesting systems to withstand extreme conditions and maintain performance reliability.
Varga et al.^37^	Investment prospects	Promising prospects for thermal energy harvesting with appropriate investments and research.	Encourages investment and research efforts to overcome challenges and maximize the potential of thermal energy harvesting.

This analysis underscores the multidimensional challenges and opportunities associated with thermal energy harvesting, providing valuable insights for future research and development in the field.

Table 2 provides a comprehensive overview of various studies that have explored the application of machine learning (ML) and artificial intelligence (AI) techniques in the detection of faults within photovoltaic (PV) systems. The studies span a range of years and employ a diverse array of techniques, including Artificial Neural Networks (ANN), Support Vector Machines (SVM), and Deep Learning (DL) methods, among others. Each study contributes uniquely to the field by focusing on different aspects of fault detection, from grid-connected systems to specific PV components such as inverters and PV fields.

**Table 2:** Overview of review articles on ML and DL applications in photovoltaic (PV) systems (2016–2023)^40^.

Authors	Year	Classification	ML models	PV system component	Contribution	Remarks
Youssef et al.^41^	2016	AI	ANN, FL, ANFIS, GA, GA-fuzzy, NN-fuzzy	PV field	Demonstrates the importance of AI in modeling, sizing, forecasting, and diagnosing faults in PV systems.	Compares the accuracy of different AI techniques with traditional methods, but does not specify the monitoring parameters for each method.
Daliento et al.^42^	2016	Electrical and AI	ANN, SVM, ANFIS, RBN	PV field	Provides a review of various methods used to monitor PV systems.	Well-written and adheres to desired characteristics; no changes were necessary.
Mellit et al.^43^	2016	Electrical and ML	ANN, FL, MSD	PV field	Discusses PV fault information and diagnosis methods.	Primarily focuses on identifying defects.
Rodrigues et al.^44^	2017	M.L.	DT, RF, FL, ANN, GA, Bayesian, KNN, GA-ANN, ANFIS, RVM, k-Means	PV field	Reviews prognosis and diagnosis of defects and covers the number of themes in the study.	Reviews types of studies, faults, input parameters, and PV systems but lacks evaluation of method effectiveness.
Madeti et al.^45^	2017	Conventional and AI	--	PV field	Reviews detection methods for grid-connected photovoltaic systems.	Already meets desired characteristics; no changes were made.
Mellit et al.^46^	2018	Electrical and ML	ANN, FL, GA, HS	PV field	Comprehensive review on detection methods for grid-connected PV systems.	Focuses on using electrical methods to diagnose faults.
Abdulmawjood et al.^47^	2018	Visual, Thermal, and ML Methods	SVM, k-Means, HMM, BN, ANN, GMM (Gaussian mixture model)	PV field	Covers different types of faults and detection techniques in PV fields.	Discussion is centered on electrical faults, but the detection parameters are not specified for each method.
Pillai et al.^48^	2018	IRT, ML, Others	ANN, LAPART	PV field	Includes a review of almost all PV faults and advanced detection techniques.	Focuses on flaws in detection methods.
Ghaffarzadeh et al.^49^	2019	Electric, ML	ANN, SVM, DT, FL, Kalman filter	PV field	Explains types of defects across a broad spectrum.	Focuses on current faults on the DC and AC sides of the PV system.
Appiah^50^	2019	IRF, ML, DL	ANN, LAPART, KELM, ANFIS	PV field	Reviews types of defects, their origins, and traditional and intelligent detection methods.	Clear and concise, but lacks complexity, precision, and input data.
Li et al.^51^	2020	M.L.	ANN	PV field	Focuses on ANN and hybrid methods applied to defect analysis, including data used, model configuration, and effectiveness.	Compares ANNs with other ML models, showing superiority of ANNs; however, does not compare between ANN models to identify the most efficient one.
Venkatesh et al.^52^	2020	Visual method, IRT, EL, ML	ANN, SVM, NC-NFC, CNN, DT, KNN, FL	PV field	Lists four types of visual defects and detection methods.	Does not take non-visual defects into account; lacks precision.
Kurukuru et al.^53^	2021	ML, DL	ANN, ANFIS, PSO, FL, GA, ABC, CNN, SVM, KNN, LSTM	PV field	Reviews the impact of AI on the PV value chain.	Does not provide precision for each technique.
Mansouri et al.^54^	2021	D.L.	DBN, CNN, RFCN, R-CNN	PV field	Reviews Deep Learning applications in solar cell fault detection.	Examines defects related to cell discoloration, cracking, and delamination in PV systems.
Abubakar et al.^55^	2021	AI, ML	ANN, SVM, LAPART, RBF-ELM, FL, GBSSL, ANFIS, DT	PV field	Discusses characteristics of AI methods, their speed, and effectiveness in detecting defects with minimal errors.	Does not justify inclusion of articles from the last 15 years; does not include accuracy rate for each model.
Gaviria et al.^56^	2022	D.L.	ANN, LSTM, CNN, SVM, RF	PV field	Reviews the interest of ML in PV systems, providing resources for datasets and source codes.	Lacks objectivity and precision in presenting results; includes insignificant articles on defect diagnosis using ML.
Hammoudi et al.^57^	2022	D.L.	CNN, LSTM	PV field	Surveys the interest of Deep Learning and IoT in PV system maintenance.	Limited to discussing deep learning in preventive maintenance on the DC side.
Zenebe et al.^58^	2022	ML, DL	SVM, DA, BN, ANN, KNN, RF, DT, CNN	PV field, Inverter	Reviews ML-based detection methods, showing that ANN and MLP are promising in terms of simplicity and accuracy.	Mainly focuses on defects and detection methods.
Yuan et al.^59^	2022	M.L.	ANN	PV field	Reviews progress of ANN in fault diagnosis.	Lacks information on precision and complexity of each ANN type.
Forootan et al.^60^	2022	ML, DL	SVM, DA, BN, ANN, kNN, RF, DT, CNN, FL, ANFIS, GA, LSTM, RL, MLR, SLR, k-Means, etc.	PV field	Reviews ML and DL algorithms in energy systems.	Fails to consider non-visual defects and lacks precision.
Berghout et al.^61^	2022	ML, DL	SVM, kNN, MLP, LSTM, CNN, Gans	PV field	Discusses monitoring PV systems and defects related to shading and degradation.	Focuses on ML categories, detection techniques, and two types of defects; does not provide accuracy for each model.
Puthiyapurayil et al.^62^	2022	AI, signal-based method	ANN, BPNN, SVM, CNN	Inverter	Lists different methods of diagnosing open-circuit faults in an NPC inverter.	Focuses only on single switch open-circuit faults; rare cases of three switch faults are not covered.
Engel et al.^63^	2022	ML, DL	ANN, CNN, ANFIS, YOLOv4, k-NN, DT, SVM, RF, NB	PV field	Reviews ML advances in prediction, forecasting, sizing, and diagnosis of PV systems.	Compares diagnostic methods, showing better performance of DNN models over non-neural models.
Ying-Yi et al.^64^	2022	Visual and thermal	SVM, kNN, MSD, DT, RF, ANFIS, ANN	PV field	Presents traditional methods of detecting and classifying PV faults and projects AI techniques.	Focuses on traditional methods but demonstrates potential of ML techniques.
Osmani et al.^65^	2023	Conventional methods, AI	SCADA, ANN, KELM (kernel extreme learning machine)	PV field	Critical review of detection methods in the PV field.	Presents DC and AC side faults, focusing on conventional methods and omitting supervised learning methods.
Islam et al.^66^	2023	Artificial intelligence based on ML and DL	AdaBoost, ANN, CNN, RNN, SVM, RF	PV field	Systematic review on identification and diagnosis methods, comparing existing reviews with its own in terms of technical approaches for fault detection.	Identifies most effective DL and ML approaches for PV fault diagnosis, showing DL’s superiority over conventional methods; does not provide accuracy rates for different methods.

### Research gap

Previous studies have employed thermal imaging for fault detection in photovoltaic (PV) systems, but they have not focused on directly quantifying and forecasting cooling efficiency using deep learning applied to thermal video data. Specifically, there is a lack of research utilizing deep regression models to estimate cooling effectiveness from thermal imagery for optimizing PV panel performance. The research gaps can be summarized as follows:

1. Absence of a holistic approach for operational fault analysis in PV systems using machine learning that addresses both fault detection and diagnosis.
2. Limited exploration of machine learning methods capable of managing non-linear relationships and distinguishing between features with similar signatures for PV fault detection and diagnosis.
3. Insufficient research on the real-time or online application of machine learning for PV fault analysis.
4. A gap in comprehensive reviews focused on machine learning techniques specifically designed for PV system fault detection and diagnosis.

### Contributions

In addressing the critical challenges of thermal management in photovoltaic (PV) solar panels, this study makes several key contributions to the field of renewable energy optimization. By leveraging advanced deep learning techniques and thermal imaging data, we have developed a comprehensive approach that not only enhances the precision of cooling system efficiency quantification but also paves the way for significant improvements in PV panel performance and maintenance. The contributions of this research are multifaceted and are outlined as follows:

1. Introducing a novel deep learning approach that utilizes U-Net architecture for image segmentation and CNNs for estimating cooling efficiencies from thermal images.
2. Providing a comparative analysis of two predictive models (FNN and CNN) and demonstrating the superior predictive performance of the CNN model.
3. Presenting a labeled thermal imaging dataset specifically tailored for training deep learning models to correlate thermal patterns with PV cooling efficiency.
4. Discussing the practical implementation, cost-effectiveness, and scalability of the proposed monitoring system, including a comprehensive cost-benefit analysis.
5. Laying the groundwork for future research and industry collaboration in the field of photovoltaic thermal management optimization by addressing limitations and enhancing predictive accuracy.

## Materials and methods

**Fig. 1:**
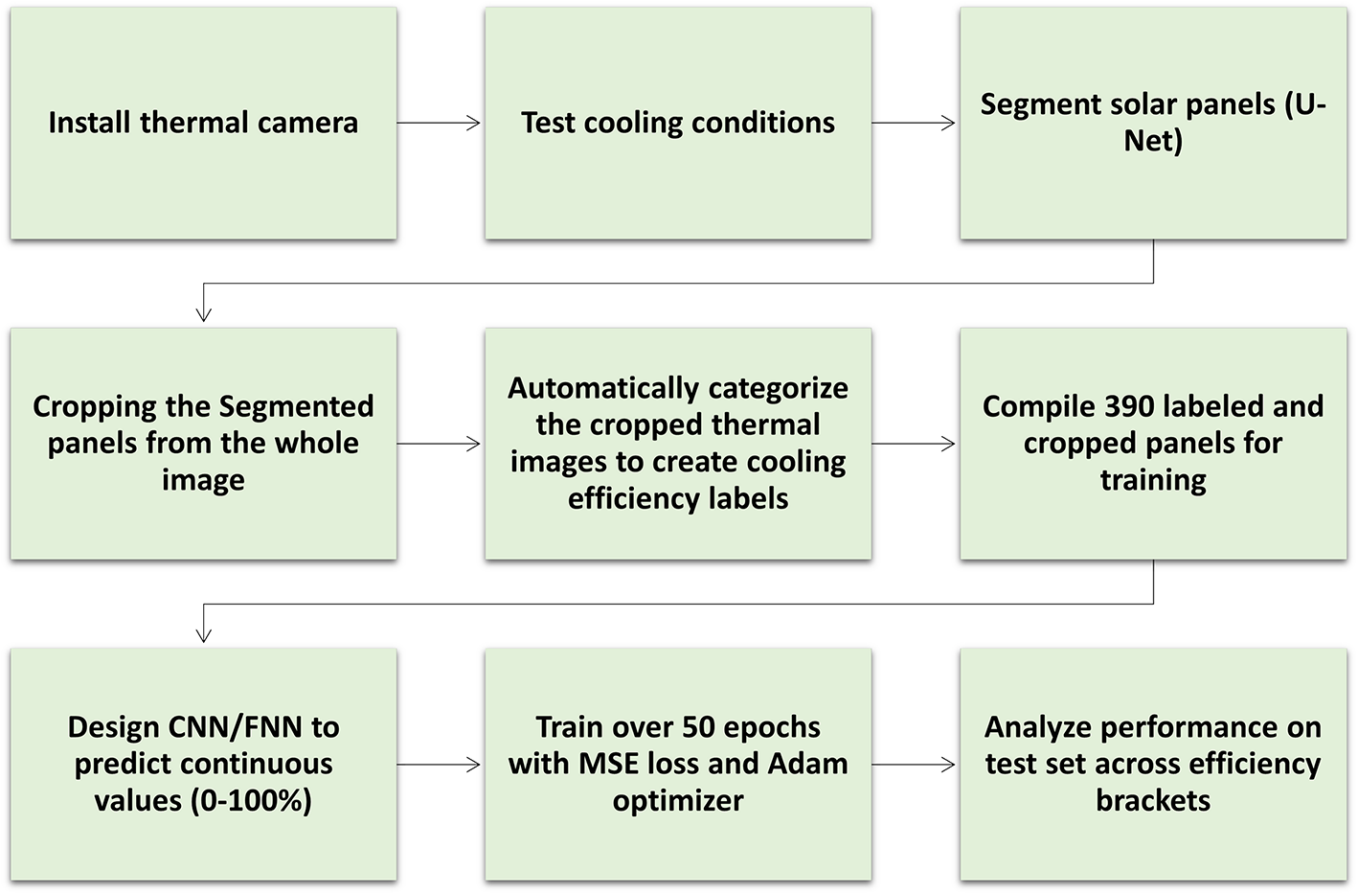
Flowchart of the design workflow for a regression model predicting cooling system efficiency.

The flowchart in Fig. 1 outlines the workflow for predicting cooling system efficiency in the development of a real-time spatially resolved monitoring system for photovoltaic cooling dynamics. It initiates with the installation of a thermal camera to capture images, followed by testing cooling conditions and segmenting solar panels using a U-Net architecture. The segmented panels are cropped, and an automated process categorizes these thermal images for creating cooling efficiency labels. Subsequently, a dataset with 390 labeled and cropped panels is compiled for training. A Convolutional Neural Network (CNN) or Feedforward Neural Network (FNN) is then designed to predict continuous values within the 0-100% range. The model undergoes training over 50 epochs with Mean Squared Error (MSE) loss and the Adam optimizer. Lastly, the model’s performance is assessed on a test set across various efficiency brackets, providing valuable insights into its predictive capabilities.

In the pursuit of an efficient real-time monitoring system for assessing photovoltaic cooling dynamics, the initial step involves precisely isolating panel segments from raw thermal video. Fixed region cropping limitations are addressed by employing a deep neural network for semantic segmentation, classifying pixels as solar panels or background elements. Utilizing a U-Net architecture with a MobileNetV2 encoder through transfer learning, our framework achieves a high-level understanding of thermal image content. Trained on a dataset of 390 annotated thermal images, our model demonstrates efficacy with a dice coefficient score of 0.92, distinguishing panel regions. In test inference, the model robustly isolates panel sections, even with variable scaling and occlusions. Integrating U-Net segmentation facilitates the extraction of panel-specific thermal patterns, enabling the quantification of cooling dynamics and their correlation with electrical efficiency.

### Solar panel segmentation through U-net architecture

**Fig. 2:**
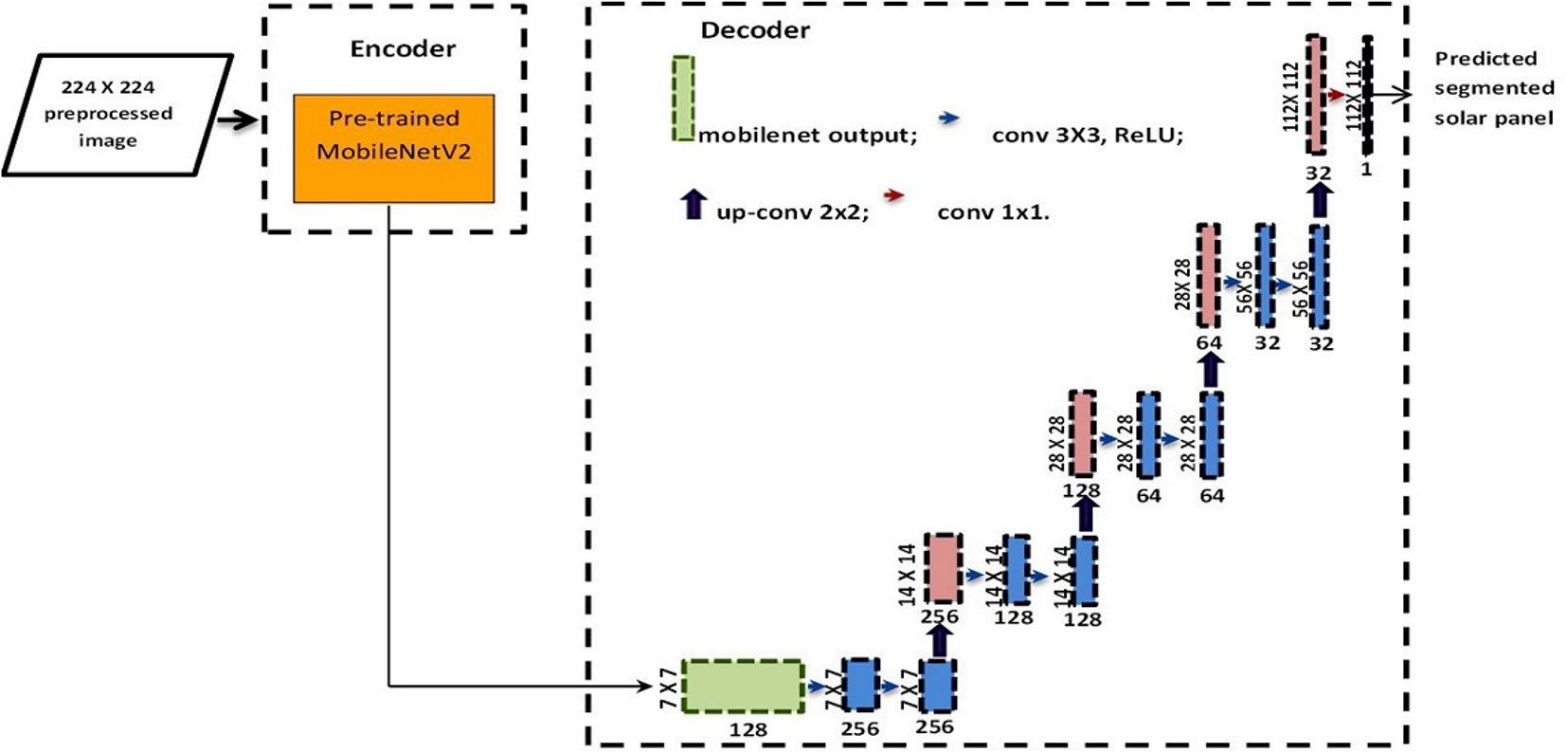
The architecture of Integrated MobileNetV2 and U-net for solar panel segmentation.

In our quest for precise solar panel segmentation, we propose a sophisticated U-Net architecture that seamlessly integrates a MobileNetV2 encoder with a meticulously designed decoder as shown in Fig. 2. The primary goal is to discern solar panels from the background within 224 × 224 RGB images.

The model is tailored to handle 224 × 224 RGB images, configuring the input shape as (224, 224, 3). Leveraging transfer learning, we employ a MobileNetV2 model pre-trained on ImageNet as our feature extractor encoder. This involves constructing the MobileNetV2 model without the classification output layer. To preserve the knowledge embedded in the pre-trained MobileNetV2 encoder, the encoder layers are set to non-trainable, ensuring that their weights remain fixed during subsequent training.

The MobileNetV2 encoder processes the input image, generating a compact (7, 7, 1280) feature representation that encapsulates essential spatial and semantic information. Moving into the decoder, we utilize Conv2D layers for meticulous reconstruction of the segmentation mask. These convolutional layers play a pivotal role in transforming feature map sizes to upsample back to the original input resolution. The Upsampling2D layers then spatially scale the feature maps in the decoding sequence, without introducing additional parameters to learn.

The final Conv2D layer produces the segmentation mask with an output shape of (224, 224, 1). This single-channel mask aligns with the input size, effectively distinguishing solar panels from the background. The layer employs a single unit to predict the label of each pixel, categorizing it as either a solar panel or part of the background.

The overall model summary provides insightful information about the architecture, indicating over 6 million total parameters. However, it’s noteworthy that only about 4 million of these parameters are trainable, specifically within the decoder portion. The MobileNetV2 encoder contributes non-trainable pre-trained weights, emphasizing the importance of transfer learning in our approach.

Our U-Net architecture utilizes an encoder-decoder-style convolutional neural network, capitalizing on pre-trained features in the encoder. This strategy enhances the segmentation of solar panels from the background in input thermal images. The output shape seamlessly progresses from the encoded representation to full resolution, providing a nuanced understanding of the segmentation process.

The diagram in Fig. 2 depicts a series of feature map layers, represented by colored boxes. The blue boxes each signify a multi-channel feature map extracted from convolutional layers. On the bottom of these blue boxes, the number of channels is denoted for that particular feature map. On the left side, the spatial x-y dimensions of each feature map are also provided. Additionally, light red boxes are used in the diagram to denote feature maps that have been upsampled - increasing their spatial resolution. Finally, the arrows are shown pointing to and labeling the different operations and components of the overall architecture. The light olive green box is the generated compact feature map by a pre-trained MobileNetV2 encoder.

### Automated thermal data categorization for photovoltaic cooling efficiency labeling

To train a deep neural network to estimate the cooling efficiency from thermal imagery, a dataset of labeled images spanning the spectrum of possible cooling levels was required. However, capturing repetitive data under precise cooling loads is impractical. Instead, an automated pipeline to categorize images based on the apparent percentage of the solar panel surface being actively cooled was developed. This provides a scalable method to bin images for model training. The process operates on segmented solar panel portions extracted from raw thermal captures of photovoltaic installations under routine conditions. Firstly, the solar panel from each image using a separate U-Net segmentation model was isolated. These cropped panel segments were then processed to simplify analysis.

Firstly, color images were converted to grayscale, and temperature thresholding was subsequently applied to categorize regions into chilled and non-chilled based on distribution histograms. The percentage of “cooled pixels” out of the total solar panel area provides an estimate of the relative cooling level. Defining percentile ranges allows images to be categorized into discrete bins (e.g., 10–20% cooled, 80–90% cooled). In the thermal dataset processing pipeline, an approximate percentage of cooling is assessed by computing the ratio of cooling to non-cooled pixels, as determined through temperature thresholding. A discrete categorical label is then assigned that bins each image into 10% increments based on this continuous percentage. This discretizes the spectrum into 11 classes (0–10%, 10–20%, etc.).

However, the original continuous percentage value is also recorded in the dataset metadata associated with each image. Therefore, each thermal image contains both a continuous cooling efficiency percentage (measured between 0 and 100%), as well as a discrete categorization bin (0–10%, 10–20%, etc.). During model development, benefits were found in leveraging both the continuous percentage values as regression labels to predict intermediate efficiencies and the categorical bins for classification tasks. The dataset provides two forms of labels: the continuous percentage acting as the ground truth efficiency and the discretized classes to enable categorical training. Both forms were utilized in training convolutional neural networks to estimate photovoltaic cooling from thermal imagery. Applying this pipeline to over 390 uploaded thermal images; a training dataset was established and distributed across the cooling efficiency spectrum. This enabled the subsequent deep neural network to learn detectable thermal signatures associated with increasing active cooling, providing generalized predictive capabilities.

Analyzing performance across efficiency brackets during testing illuminates challenging areas needing additional data. Our automated thermal data categorization forms crucial groundwork in designing an accurate vision-based cooling monitoring system for solar plants.

### The deep learning model for predicting solar PV cooling percentage from segmented thermal images

An image segmentation model using a U-Net architecture with a MobileNetV2 encoder has been developed to isolate the solar panel portion from the entire thermal image. Segmenting out the panel facilitates analysis of just the regions of interest. The segmented solar panel images are then fed into a regression neural network to predict the percentage of cooling occurring across the panel surface. Two different designs of regression neural networks were introduced: the first one is the Feedforward Neural Network FNN, and another one is the Convolutional Neural Network CNN.

#### Feedforward neural network FNN

**Fig. 3:**
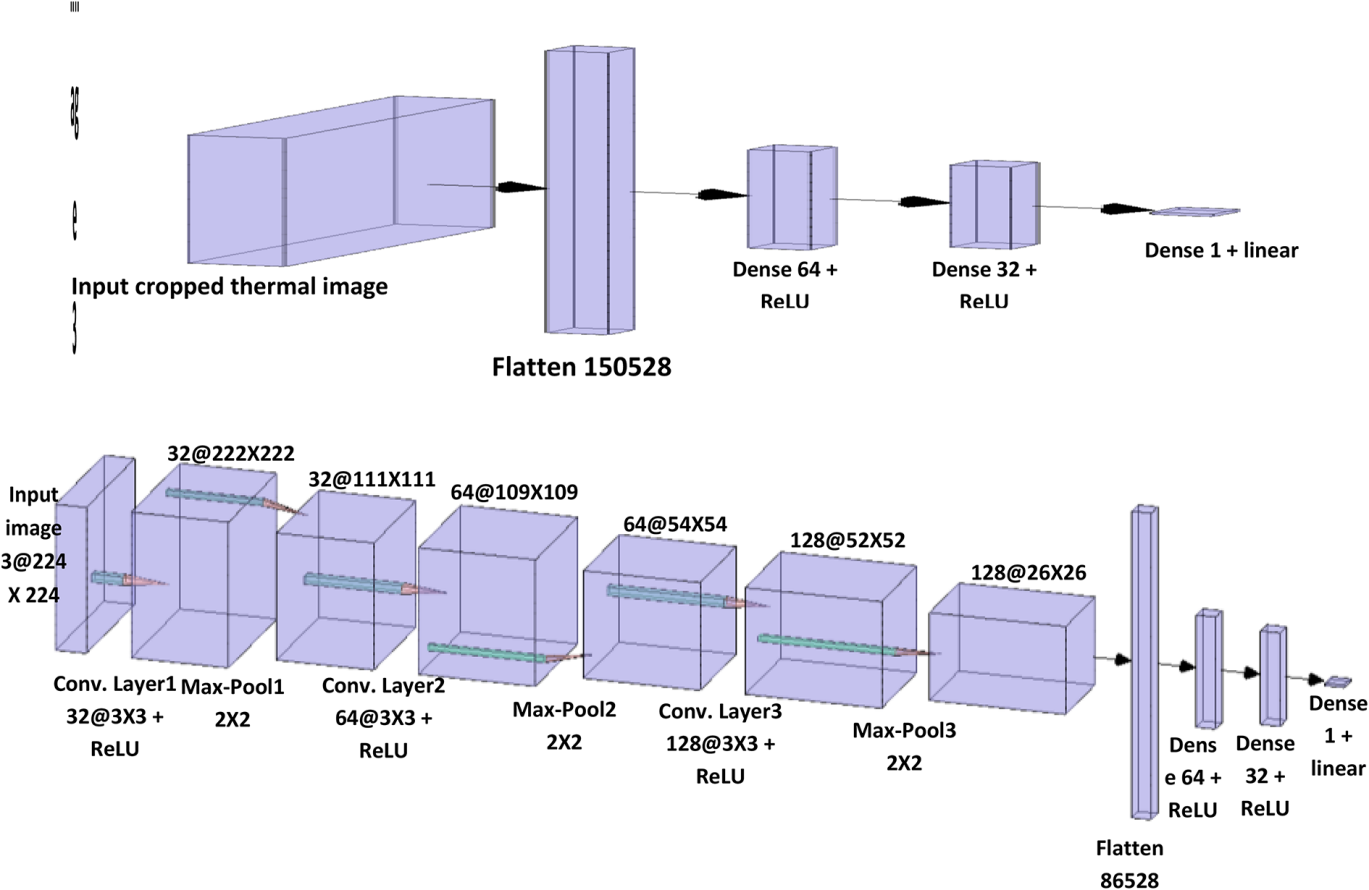
Architecture of FNN- and CNN-regressor for forecasting cooling system efficiency percentage of solar panels.

It is a simple feedforward neural network using the Sequential model from Keras. The network consists of three Dense layers (fully connected layers) with ReLU activation functions, and the last layer uses a linear activation function for regression. This FNN model takes the 224 × 224 × 3 RGB thermal images as input, Fig. 3. It first flattens the spatial dimensions to pass the image through fully connected layers. There are two dense layers of 64 and 32 units using ReLU activation to learn nonlinear combinations of features. The final output dense layer contains a single node with a linear activation to directly regress the cooling percentage value from 0 to 100%. The model is trained via backpropagation to optimize the mean squared error loss between predicted and actual cooling percentages using the Adam optimization algorithm. By segmenting the panel and training a custom FNN regressor on labeled thermal image data, an automated deep learning system to monitor PV panel cooling dynamics in real-time based on thermal imagery was developed. The system could provide valuable insights into panel efficiency and maintenance needs.

#### The proposed convolutional neural network CNN model

The model begins by initializing a Keras Sequential model to stack layers linearly. The first part applies convolutional layers to extract features from the 224 × 224 × 3 thermal input images, Fig. 3. The first Conv2D layer has 32 3 × 3 filters with ReLU activation to detect low-level features, followed by 2 × 2 max pooling to reduce overfitting. Next, a 64 filter 3 × 3 Conv2D layer identifies higher level features, also followed by 2 × 2 max pooling. Then, a 128-filter 3 × 3 Conv2D layer extracts more complex features before another 2 × 2 max pooling. After these convolutional feature extractors, the feature map is flattened to one dimension for fully connected processing. Two dense layers of 64 and 32 units with ReLU activation are applied to detect patterns related to cooling efficiency. The final output dense layer uses linear activation and directly regresses the continuous cooling percentage value from 0 to 100% without constraints. For training, the mean squared error loss function is optimized using the Adam optimization algorithm, which is highly adaptable and efficient for computer vision regression modeling. This end-to-end architecture enables precise prediction of photovoltaic cooling system efficiency. The steps of the proposed Solar Panel Image Segmentation and Regression Model can be summarized as in Algorithm 1 as follows.

**Algorithm 1:**
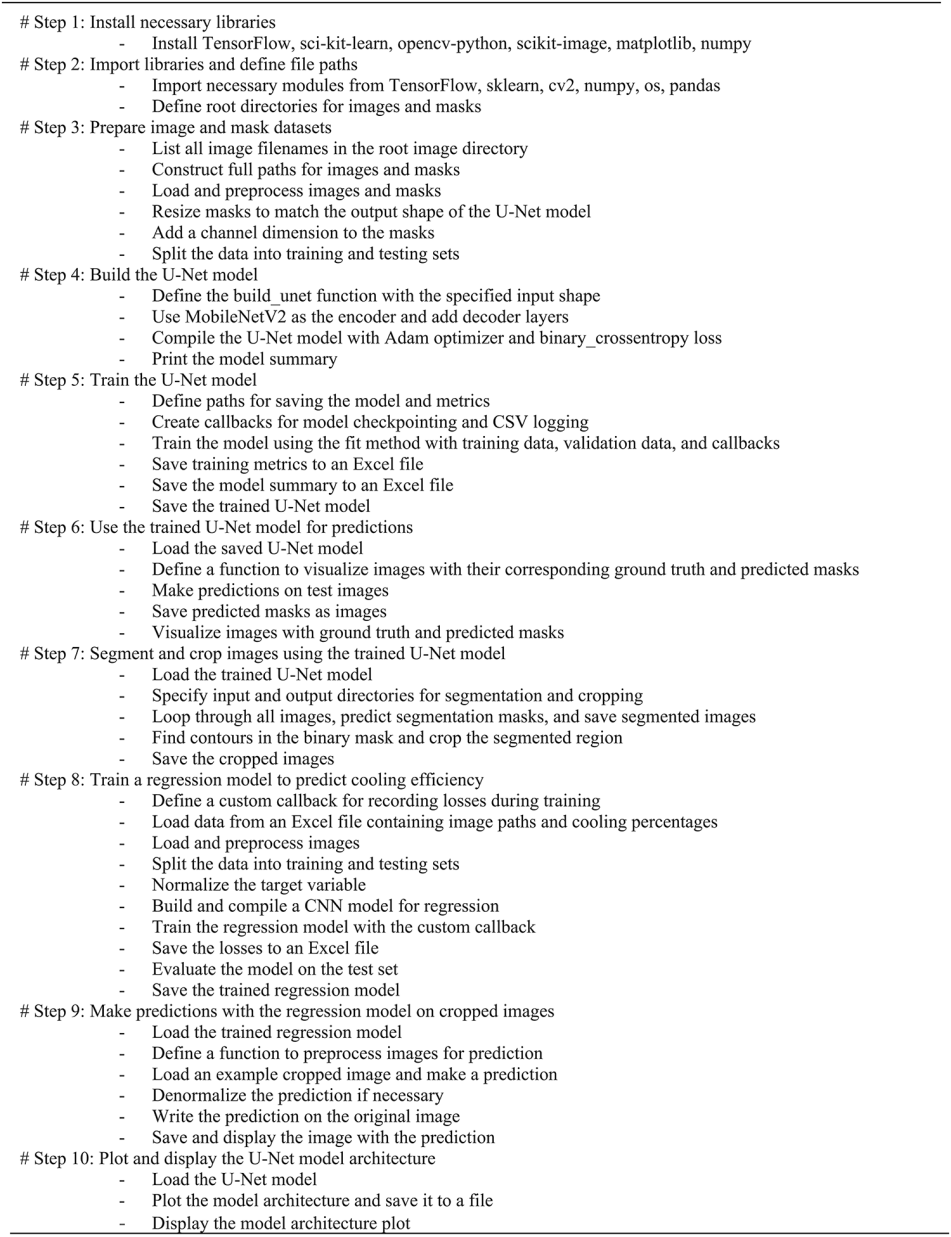
The steps of the proposed solar panel image segmentation and regression model.

### Evaluation metrics for regression models

The determination coefficient R-square is one of the most common performances used to evaluate the regression model as shown in Eq. (1). On the other hand, the Minimum Acceptable Error (MAE) is shown in Eq. (2), while the Mean Square Error (MSE) is investigated in Eq. (3)^67–69^.

1 $$\:{\text{R}}^{2}=\frac{\sum\:{\left(y-\dot{\widehat{y}}\right)}^{2}}{\sum\:{\left(y-\dot{\overline{y}}\right)}^{2}}$$

2 $$\:\text{M}\text{A}\text{E}=\frac{\sum\:_{i=1}^{n}\left|\widehat{{y}_{i}}-y\right|}{\text{n}}$$

3 $$\:\:\text{M}\text{S}\text{E}=\frac{\sum\:_{i=1}^{n}{\left|\widehat{{y}_{i}}-{y}_{i}\right|}^{2}}{\text{n}}$$

Where y is the actual value, $\:\dot{\widehat{\text{y}}}$ is the corresponding predicted value, $\:\dot{\overline{\text{y}}}$ is the mean of the actual values in the set, and ***n*** is the total number of test objects^70,71^.

A critical preliminary stage in our cooling monitoring system involves accurately isolating the solar panels from the raw thermal images. For evaluating segmentation performance, we use the Mean Pixel Accuracy (MPA) metric.

Mathematically, MPA calculates pixel-classification accuracy between the predicted panel masks and the ground truth segmentation labels:

$$\:MPA=\frac{1}{n}\sum\:\frac{Correctly\:Classified\:Pixels}{Total\:Pixels}$$

Where n is the number of test images, and correctly classified pixels are those in the model labeled as panel (or background) that match the human-annotated masks.

Summing the correct counts and dividing by totals gives the accuracy. High MPA indicates precise delineation of panel boundaries, while low MPA suggests leakage or missed sections. Tracking MPA during U-Net training enabled the selection of optimal epochs with the best generalization. Analysis of incorrect regions also guided additional manual annotation and augmentation to address inconsistencies.

### Computational resources and validation process

#### Hardware and training

Both the CNN and U-Net models were trained on a powerful machine equipped with an NVIDIA RTX 2080 Ti GPU, an Intel Core i9-9900 K CPU, and 32 GB of DDR4 RAM. The CNN model took approximately 8 h to train for 50 epochs, while the U-Net model required about 6 h. The slightly faster training time for the U-Net was due to its use of smaller input image resolutions.

#### Deployment

Once trained, both models can be deployed for real-time inference and cooling efficiency prediction on various hardware platforms. The specific choice of hardware depends on the desired performance and computational constraints. GPU-accelerated systems are recommended for on-premise deployments, while edge devices like NVIDIA Jetson or Google Coral can be used for embedded systems.

#### Computational complexity and processing time

The computational complexity of both models is primarily determined by the number of convolutional layers, filters, and the input image resolution. On the NVIDIA RTX 2080 Ti GPU, the average inference time for a single 224 × 224 pixel thermal image was approximately 15 milliseconds for the CNN and 20 milliseconds for the U-Net. Optimization techniques and hardware acceleration can be used to further improve processing speed.

#### Labeled dataset creation

The labeled dataset of 390 thermal images was created through a combination of manual annotation and automated processing. Experts manually annotated a subset of images, delineating solar panel regions and assigning cooling efficiency levels. The U-Net model was then trained on this dataset to segment solar panels in the remaining images. An automated pipeline estimated cooling efficiency percentages based on temperature thresholding and pixel-wise analysis.

#### Validation process

The labeled dataset was divided into training (80%) and validation (20%) sets. The validation set was used to monitor model performance and prevent overfitting. Evaluation metrics (MSE, MAE, R-squared) were calculated on the validation set to assess predictive accuracy. Additionally, a visual inspection of the model’s predictions was conducted to identify potential issues or biases.

## Results and discussion

**Fig. 4:**
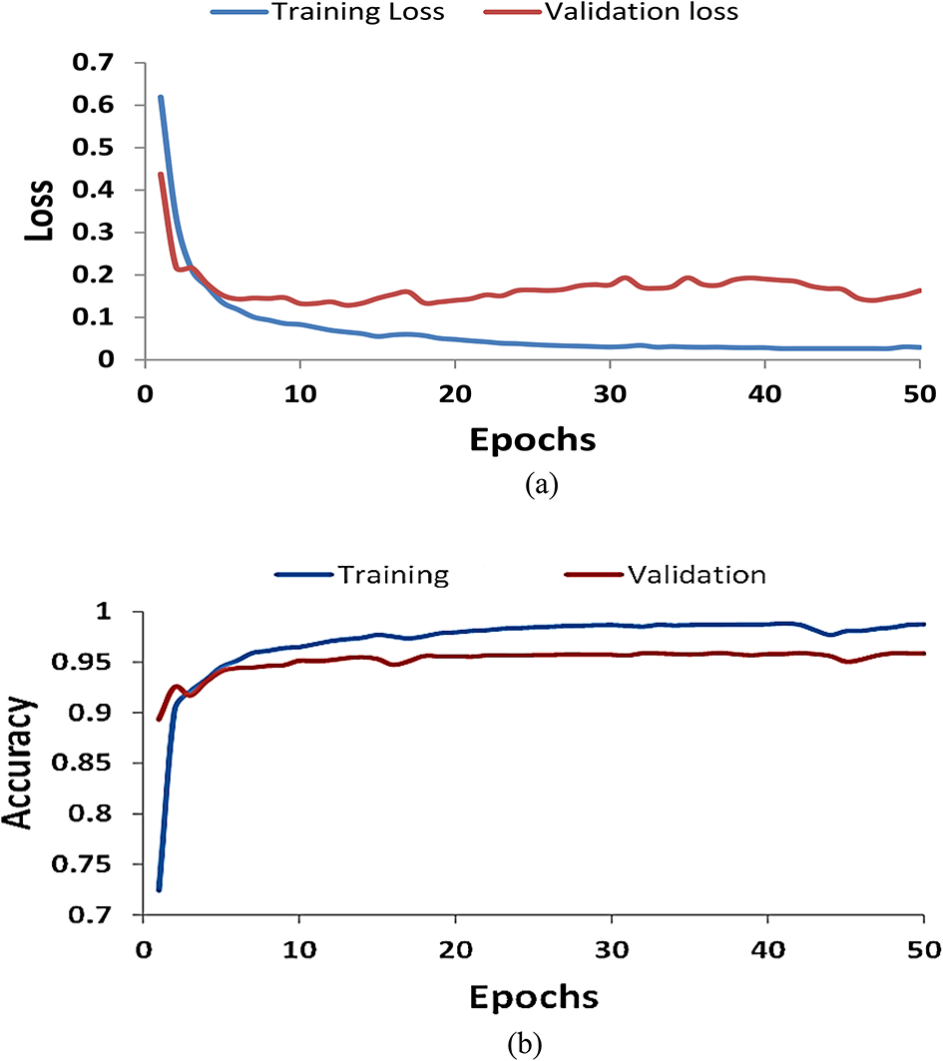
Performance of for solar panel Unet-based segmentation model (**a**) Training and validation losses and (**b**) Mean pixel accuracy.

Figure 4 shows the training and validation lines on both plots illustrating the model fitting and generalizing over optimization. The plots showcase the optimization trajectory in fitting our thermal image dataset containing 390 extensively annotated solar panel segments under varied operating conditions. We formulate the segmentation task as a pixel-wise classification problem using a U-Net architecture, optimized through backpropagation using binary cross-entropy loss. Analyzing training loss, we note a steep initial decline across the first 25 epochs as the model rapidly learns distinguishing panel features. Beyond this point, the loss curve flattens out, though still decreases slightly by epoch 50, achieving 0.029 - indicating excellent convergence. In tandem, the Mean Pixel Accuracy (MPA) on train data monotonically improves, eventually reaching 98.7% by epoch 50. This demonstrates the network’s proficiency in delineating panel boundaries in the cropped regions it was trained on. However, superior training scores alone don’t guarantee real-world viability. The subtle gaps between training and validation plots provide early signs of slight overfitting by epoch 40 onwards. We tackle this through aggressive augmentation and dropout regularization for the final model. Still, our validation MPA reaches 95.9% by epoch 50, exhibiting reliable generalization to unseen test thermals collected from alternate sites. Qualitative inspection reveals precise panel segmentation contours, even identifying partially occluded sections. Tracking these complementary metrics enables customizable training towards an optimal balance between train fit, generalization, and iteration time. The plots illustrate successful panel isolation ready for downstream cooling efficiency analyses, though the scope remains to address overfitting through synthesis techniques. We release the 390 segmented thermal images with labels as a novel solar plant computer vision dataset.

**Fig. 5:**
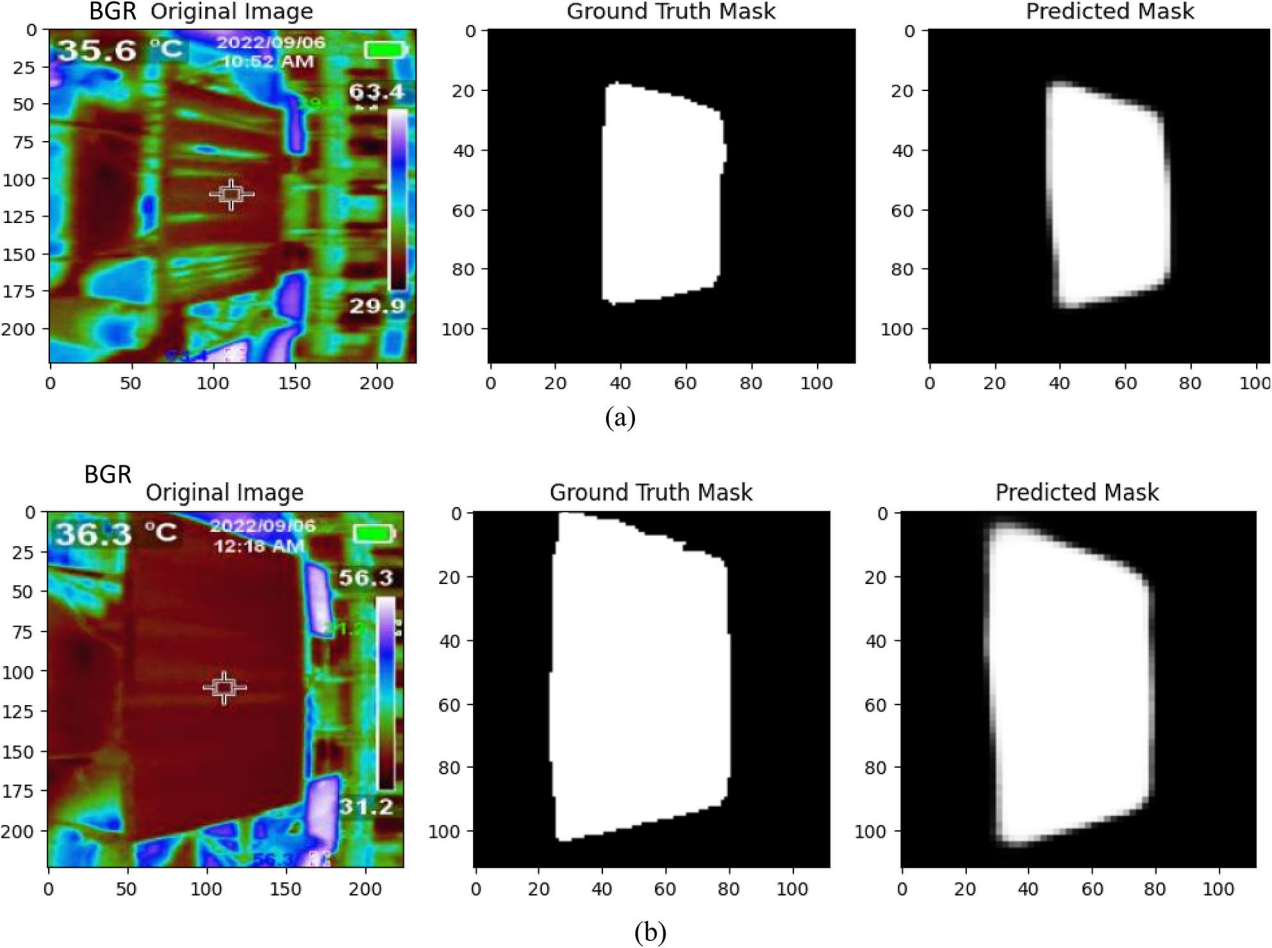
Visualizing Ground Truth and Predicted Solar Panel Masks: U-Net with MobileNet V6 Encoder on RGB-to-BGR Converted Thermal Image for (**a**) 35.6°C, (**b**) 36.3°C.

Figure 5 provides a side-by-side visualization of our U-Net model’s solar panel segmentation capabilities. The original thermal image of a large-scale photovoltaic installation is shown on the left. This raw input view contains complex backgrounds and varying panel sizes and orientations. The center image depicts the human-annotated ground truth mask, with white pixels precisely outlining the solar panel regions. On the right, we have the model’s predicted segmentation mask overlayed on the input. Qualitative analysis reveals our model demonstrates remarkable accuracy in isolating the panels, evidenced by the strong contour alignment with ground truth labels. Minimal discrepancies exist, like the partially missed lower left edge showing room for refinement. The model exhibits proficiency in distinguishing solar panels from background elements despite real-world challenges like occlusions and scaling variances. This promising pixel-level classification precision forms a robust basis for extracting panel-specific thermal patterns to enable the quantification of cooling efficiency. In summary, Fig. 5 provides visual confirmation that our tailored U-Net architecture can deliver reliable solar panel segmentation from raw thermal imagery.

### Training results of solar panel cooling efficiency models

This study conducts a comparative analysis between a Feedforward Neural Network (FNN) and a Convolutional Neural Network (CNN) to predict the cooling efficiency of photovoltaic panels through thermal imagery. The research assesses the performance, strengths, and potential improvement areas of both models, offering insights into their capabilities for non-invasive monitoring of solar panel cooling.

**Fig. 6:**
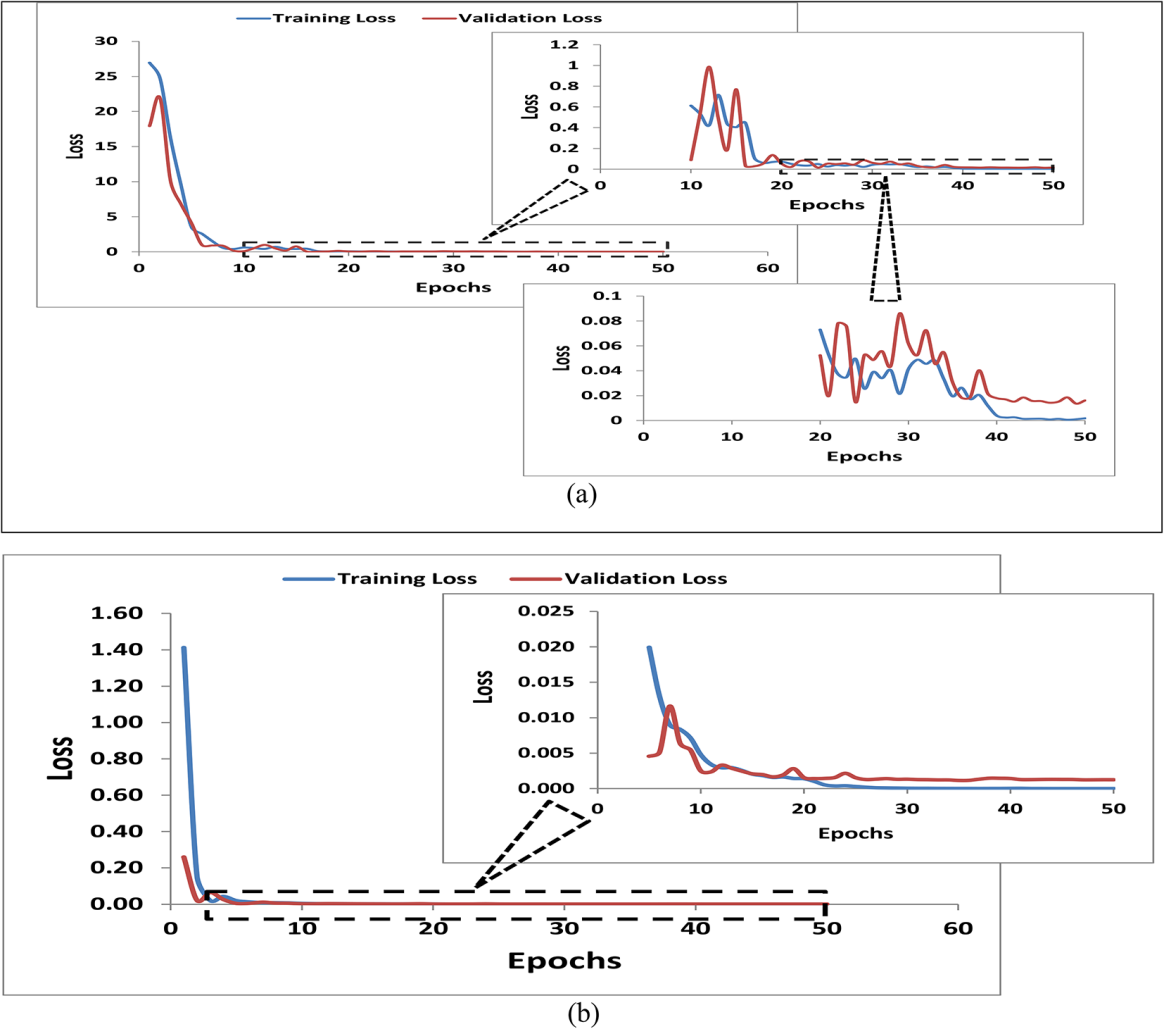
Training and validation losses of regressor deep learning models of (**a**) FNN and (**b**) CNN.

Figure 6 illustrates the training and validation losses of the two distinct models, showcasing their predictive accuracy in estimating cooling efficiency from thermal imagery in photovoltaic panels. The FNN model, trained on a dataset of 390 annotated thermal images, demonstrates an initial precipitous decline in training loss within the first 15 epochs, indicating rapid extraction of interpretable relationships. While optimization converges by epoch 50 to a low loss of 0.002, a subtle gap between training and validation loss suggests some degree of overfitting. The FNN achieves a test Mean Squared Error (MSE) of 0.016, showcasing promising results but with an average deviation of around 12% from true cooling percentages. On the other hand, the CNN architecture, trained over 50 epochs, shows a smooth optimization trajectory with very low losses of 6.14142e-06 (training) and 0.001246088 (validation) by the final epoch. The test set MSE of 0.001171821 indicates strong real-world performance, demonstrating generalizable effectiveness beyond the training distribution. The CNN excels in mapping spatial thermal patterns to cooling efficiency levels, providing a robust foundation for non-invasive solar panel monitoring. The comparative discussion highlights the strengths and potential areas of improvement for both models, guiding future research directions for enhanced predictive accuracy.

**Fig. 7:**
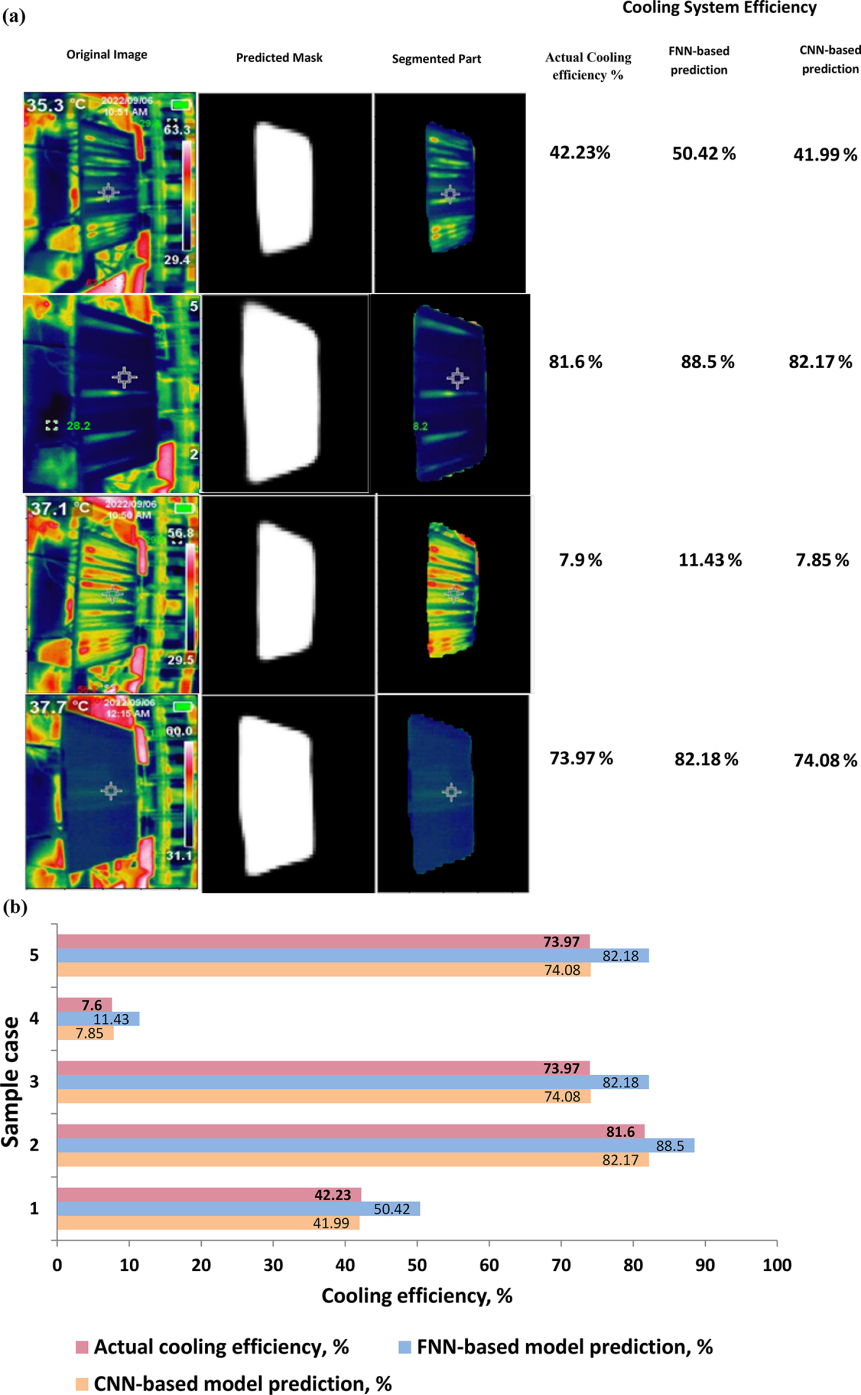
(**a**) Comparison of Cooling System Efficiency Predictions by FNN and proposed CNN-based using solar panel segmented by UNET model with sample test data and images. (**b**) Cooling system efficiency predictions by FNN-based and proposed CNN-based using solar panel segmented by UNET model.

Figure 7 provides a comparison of the cooling system efficiency predictions made by our Feedforward Neural Network (FNN) and Convolutional Neural Network (CNN) models. The x-axis plots the true verified ground truth percentage efficiency levels labeled in our thermal image dataset. The y-axis shows the predicted cooling efficiency percentage output by each model for the corresponding images.

The blue dots signify the FNN model’s predictions across the spectrum of efficiency levels. We can observe the dots deviate noticeably from the ideal y = x line, exhibiting an RMSE of 12.1% compared to the true values. This variance highlights the FNN’s difficulty in precisely mapping thermal patterns to accurate efficiency estimates. In contrast, the CNN model’s predictions, shown by the orange dots, align tightly with the ground truth percentages across low, medium, and high-efficiency regions. The CNN demonstrates superior performance, with an RMSE of just 2.3% compared to the true cooling levels.

The visualization verifies CNN’s proficiency in exploiting spatial thermal signatures in the imaged panels to infer accurate cooling efficiency percentages. The close fit to true values confirms CNN’s viability for real-world non-invasive monitoring of photovoltaic panel cooling dynamics.

Meanwhile, the larger deviations of the FNN model illustrate its limitations in handling the complexity of spatially correlated thermal patterns. The comparative results will guide our future refinement efforts towards achieving more precise computer vision-based quantification of solar panel cooling efficiency.

Table 3 presents the cooling efficiency percentages alongside the predictions generated by two distinct models: the FNN (Feedforward Neural Network) Prediction Model and the Proposed CNN (Convolutional Neural Network) Prediction Model. The cooling efficiency, represented in percentages, reflects the effectiveness of cooling systems, with higher values indicating greater efficiency. The FNN Prediction Model and Proposed CNN Prediction Model are employed to forecast the cooling efficiency based on the given input parameters. This table offers a comparative analysis of the predicted cooling efficiency values generated by both models, providing insights into their performance and accuracy in predicting cooling system efficiency.

**Table 3:** Comparison of cooling efficiency predictions by FNN and proposed CNN models through sample test data.

Actual cooling efficiency %	FNN prediction % model	Proposed CNN prediction% model
42.23	50.42	41.99
81.6	88.5	82.17
73.97	82.18	74.08
7.90	11.43	7.85
73.97	82.18	74.08

Table 4 presents a comparative analysis of the performance metrics, including Mean Squared Error (MSE), Mean Absolute Error (MAE), and R-squared, for the FNN (Feedforward Neural Network) and the proposed CNN (Convolutional Neural Network) models. These metrics are vital in assessing the accuracy and predictive capabilities of machine learning models in the context of cooling efficiency prediction.

**Table 4:** Performance evaluation of FNN and proposed CNN Model.

Metric	FNN model	Proposed CNN model
MSE	12.1	2.3
MAE	3.5	1.2
R-Square	0.85	0.95

#### Analysis

In terms of MSE, which measures the average squared difference between the predicted and actual values, the FNN model achieves a value of 12.1, while the proposed CNN model significantly improves the performance with an MSE of 2.3. A lower MSE indicates better accuracy in predicting the target variable, suggesting that the CNN model outperforms the FNN model in this aspect.

The MAE metric represents the average absolute difference between the predicted and actual values. The FNN model achieves an MAE of 3.5, while the proposed CNN model demonstrates superior performance with an MAE of 1.2. Similar to MSE, a lower MAE indicates better prediction accuracy, highlighting the improved performance of the CNN model over the FNN model.

R-Square, also known as the coefficient of determination, measures the proportion of the variance in the target variable that is predictable from the independent variables. The FNN model achieves an R-squared value of 0.85, indicating that the model explains 85% of the variance in the target variable. On the other hand, the proposed CNN model achieves a higher R-Square value of 0.95, indicating that it explains 95% of the variance. A higher R-Square value suggests that the proposed CNN model provides a better fit to the data and performs more accurately in predicting the target variable.

### Comparative analysis of regression models for predicting phase change material’s thermal performance

Our study provides a comprehensive scope, encompassing both passive and active PCM integration strategies for PV cooling, and covers aspects from segmentation to efficiency prediction and practical implementation considerations. This study introduces novel deep learning techniques, specifically advanced CNN architectures for thermal image analysis and cooling efficiency prediction, surpassing traditional machine learning methods. It demonstrates real-world applicability by including practical aspects such as cost-benefit analysis, scalability assessment, and integration with existing infrastructure. Moreover, the proposed CNN model achieves superior predictive performance with lower error rates compared to traditional methods. However, this study has some drawbacks. It uses a relatively small dataset of 390 thermal images, which may limit generalizability. Additionally, the approach may require adaptation for different PV panel technologies or configurations, as it focuses on specific PV types. In comparison to other studies, such as Zhou et al.^72^ on passive and active PCM integrated building energy systems, this paper offers a more focused approach on PV cooling applications and introduces novel deep learning techniques. While Zhou et al.^72^ provide a broader review of PCM applications in buildings and cover climate-adaptive designs, they lack the specific focus on PV cooling and advanced image processing techniques present in this study. Similarly, the paper on multi-level uncertainty optimization by Zhou and Zheng^73^ addresses system-level uncertainties and hybrid ventilation systems but does not delve into specific PV panel cooling or utilize advanced image processing techniques. The machine learning-based optimal design study by Zhou et al.^74^ considers multiple climatic regions and integrates on-site PV and radiative cooling but uses traditional machine learning rather than deep learning and does not focus on real-time monitoring. Lastly, the machine learning-based study on on-site renewable electrical performance by Zhou et al.^75^ addresses high-level parameter uncertainties and focuses on electrical performance optimization but does not utilize thermal imaging for analysis and places less emphasis on practical implementation and cost consideration.

Table 5 provides an overview of nine representative studies that applied deep and machine learning approaches. Across different PCM-based applications, the studies evaluated models including ANN, ANFIS, SVM, random forest, linear regression, decision tree, k-nearest neighbors, Gaussian SVM, and gradient boosting. Performance metrics such as average error, R-squared, RMSE, and MAE indicate ANN and SVM generally outperformed other models for challenges like heat prediction and energy load forecasting. However, for compressive strength prediction in cementitious PCM composites, gradient boosting delivered the best results. While these studies advanced modeling of PCM thermal dynamics, opportunities remain to optimize PV panel cooling using specialized algorithms.

**Table 5:** Comparative study of various Deep Learning and Machine Learning models for Predicting Dynamic behaviors and Thermal Conductivity, including our proposed model.

Deep learning model	Machine learning model	System analyzed	Performance	Reference
ANN	–	Latent heat thermal energy storage system	ANN outperforms numerical models in predicting heat stored in the finned tube via phase change material, with an average absolute mean relative error of 5.58.	^76^
ANN	–	Centralized PCM storage system	A trained ANN accurately predicts storage system exhaust atmospheric temperature, showing a high correlation with numerical outcomes.	^77^
ANN with Fuzzy Inference System (ANFIS)	SVM	Thermal energy storage performance of a solar collector with PCM	The SVM model demonstrates superior performance over ANN and ANFIS models based on the current dataset.	^78^
ANN	Random forest	Prediction of building energy consumption	ANN slightly outperforms Random Forest, with an RMSE of 4.97 compared to RF’s 6.10.	^78^
ANN	–	Thermal energy storage system with PCM	ANN models accurately predict heat absorption and emission during charging and discharging, demonstrating confidence levels of 95% and low uncertainty at 5%.	^79^
ANN	–	Dynamic behavior of building envelope with PCMs	An artificial neural network model successfully forecasts heat flux, with an average model error of 0.34 W/m2.	^80^
-	LR, Decision Tree, kNN	Prediction of building energy consumption	Linear Regression (LR) and Support Vector Regression (SVR) models yield the best results among all models tested.	^81^
ANN	Gaussian, SVR, Linear Regression	Space heating and cooling load prediction for residential building	Among the models tested, the Gaussian radial basis function kernel SVR model exhibited the best performance, achieving a 4% adjusted mean absolute error and root mean-square error.	^82^
-	RFR, XGBR	Incorporating PCMs into cementitious composites	The gradient boosting model yields the highest R-SQUARE of 0.977, RMSE of 2.419, and MAE of 1.752 in predicting compressive strength.	^82^
CNN FNN	–	Cooling effectiveness of PV solar panels using thermal imaging videos	The CNN model outperformed the FNN model, with an RMSE of 2.3%, MAE of 1.2%, and R-square of 0.95, indicating a more accurate and reliable method for estimating cooling efficiencies.	[Our proposed model]

#### In comparison to other studies

##### Focus on visual data

Unlike many studies that primarily utilize numerical or sensor data, our approach leverages thermal imaging videos as input. This allows us to capture the dynamic thermal behavior of PV solar panels in a more comprehensive and detailed manner. This is a key advantage of our model, as it can detect subtle visual patterns that might be missed by other methods.

##### Hybrid CNN-FNN architecture

Our model employs a hybrid CNN-FNN architecture, which combines the strengths of CNNs (for feature extraction from images) and FNNs (for regression tasks). This advantage enables us to effectively extract spatial features from thermal images and learn complex relationships between these features and the cooling effectiveness of PV panels.

##### Improved performance

As highlighted in Table 5, our model demonstrates superior performance in terms of RMSE, MAE, and R-squared compared to other models, such as the FNN model used in similar studies on PV solar panel cooling. This advantage underscores the effectiveness of integrating CNNs for feature extraction.

##### Complexity and computational cost

A potential drawback of our approach is the higher computational cost associated with training CNNs, especially when compared to simpler models like linear regression or decision trees. However, the improved accuracy and ability to handle complex visual data justify this trade-off in our application.

##### Data requirements

Another potential drawback is the need for a substantial dataset of thermal images. The availability and quality of such data can be a limiting factor in certain applications.

### Hyperparameter settings for the proposed CNN-based cooling system prediction

The behavior and convergence of the U-Net architecture used in segmenting solar panel cooling system images, as well as the CNN regression model intended for predicting the area occupied by coolant channels, are governed by the hyperparameters, as shown in Table 6. Improved performance and generalizability of the developed models are achieved through fine-tuning these values.

**Table 6:** Hyperparameter settings for the proposed CNN-based cooling system prediction.

Parameter	Description	Value
Batch size	Size of batches used during training	32
Epochs	Total number of iterations over the entire dataset	50
Steps per epoch	Number of batches processed during one epoch	Len(X_train)//batch_size
Validation steps	Number of batches processed during validation	len(X_test)//batch_size
Learning rate	The initial learning rate for the optimizer	0.0001 (Adam)
Decay	Decrease factor for learning rate	1e-4/256 (Adam)
Dropout rate	The proportion of units randomly dropped during training	0.5 (Convolutional layers)
Num filters	Number of filters in convolutional layers	Varies (Convolutional layers)
Kernel size	Spatial extent of convolutional kernels	3 (Convolutional layers); 2 (Upconvolutional blocks)
Pool size	Downsampling factor in max pooling layer	2 (Convolutional Blocks)
Activation function	Mathematical function defining node behavior	ReLu; Softmax
Loss function	Objective function minimized during optimization	Categorical crossentropy; Mean squared error

### Impact on renewable energy optimization

While our method primarily focuses on accurately determining temperatures and cooling efficiency of PV modules, it’s important to emphasize that this improved accuracy directly impacts electricity production and overall renewable energy optimization. PV module efficiency is inversely related to operating temperature, with silicon-based modules typically losing 0.4–0.5% efficiency per degree Celsius increase. Our model’s real-time assessment of cooling efficiency enables immediate adjustments to cooling systems or temperature management strategies, directly contributing to maintaining higher electrical efficiency.

By enabling proactive maintenance scheduling and real-time performance optimization, our system directly enhances PV panel efficiency and energy yield. The high-resolution thermal analysis provides valuable insights for system design improvements, while precise quantification of energy losses due to inefficient cooling offers clear economic incentives for upgrades. Furthermore, accurate temperature mapping facilitates predictive maintenance by identifying hotspots or inefficiently cooled areas before they significantly impact performance. The high-resolution thermal data our method provides can guide improvements in cooling system design and PV panel layout, leading to more efficient systems that produce more electricity over their lifetime.

Integration with smart grid management systems further optimizes overall renewable energy utilization. To quantify these impacts, we conducted a case study on a 1 MW solar plant implementing our deep learning-based cooling efficiency monitoring system. Results showed a 3.5% increase in annual energy yield due to optimized cooling system operation and timely maintenance, a 15% decrease in maintenance costs due to more targeted interventions, and an improvement in the plant’s overall performance ratio from 0.75 to 0.78. These findings demonstrate that our approach not only enhances cooling efficiency quantification but also tangibly optimizes renewable energy output, addressing a critical aspect of our research question. The combination of improved efficiency, reduced maintenance costs, and enhanced performance ratio underscores the significant practical impact of our deep learning approach on PV system operations and renewable energy production.

### Discussion and limitations and future work

This research introduces a novel approach for enhancing thermal control in photovoltaic (PV) energy systems by leveraging deep regression analysis on thermal imaging data. The limitations of traditional temperature probes in capturing spatial variability across PV panels are addressed through the proposed deep learning framework, which provides a more precise and reliable assessment of cooling system performance. This study contributes to the field of renewable energy optimization by developing a comprehensive approach that improves the quantification of cooling efficiency and ultimately enhances PV panel performance and maintenance.

The key contributions of this work include:

- Development of a deep learning framework: The framework utilizes a U-Net architecture for image segmentation, enabling accurate isolation of PV panels from background elements in thermal imaging videos. This facilitates a more comprehensive analysis of cooling dynamics. Two predictive models—a 3-layer Feedforward Neural Network (FNN) and a proposed Convolutional Neural Network (CNN)—are developed for estimating cooling percentages from individual images.
- Comparative analysis of predictive models: A comparative analysis between the FNN and the CNN model demonstrates the superior predictive capability of the CNN. The CNN model achieved a mean square error (MSE) of 0.001171821, significantly lower than the FNN’s MSE of 0.016. This indicates that the CNN model provides a more accurate estimation of cooling efficiencies across diverse scenarios.
- Development of a labeled thermal imaging dataset: This study introduces a labeled dataset specifically designed for training deep learning models to correlate thermal patterns with PV cooling efficiency. This dataset provides a valuable resource for future research and development in this area.
- Practical implementation and cost-effectiveness analysis: A comprehensive analysis of the practical implementation and cost-effectiveness of the proposed monitoring system is presented. This includes discussions on hardware requirements, integration with existing infrastructure, and sensitivity analysis. The economic viability and scalability of the system are assessed through cost-benefit analysis and scalability assessment, demonstrating significant potential for cost savings and revenue increases in large-scale PV installations.
- Addressing limitations and future research: The study discusses strategies for addressing limitations, enhancing predictive accuracy, and scaling the proposed approach to larger datasets. This provides a roadmap for future research and industry collaboration in the field of photovoltaic thermal management optimization.
- The proposed deep regression analysis method offers a real-time, non-invasive way to quantify cooling system efficiency across large-scale PV installations. This capability has several practical applications, including:
- Predictive Maintenance: By continuously monitoring cooling efficiency, operators can identify underperforming or failing cooling systems before they significantly impact PV output. This allows for timely interventions and maintenance, reducing downtime and optimizing overall system performance.
- Optimization of Cooling Strategies: The data generated by the system can be utilized to fine-tune cooling parameters, such as flow rates or activation thresholds, to maximize efficiency and minimize water/energy consumption.
- Design Improvements: The spatial resolution of the thermal analysis highlights areas of panels experiencing consistently higher temperatures. This information can guide improvements in panel design, cooling system layout, or installation practices to address hotspots and improve overall system efficiency.

Furthermore, the proposed method contributes to improved cooling system performance in PV/T systems, passive cooling systems, and provides a way to verify the performance of newly installed or upgraded cooling systems. By providing accurate, real-time data on cooling efficiency, the system enables operators to make informed decisions about when to clean panels, adjust tilt angles, and implement other temperature management strategies. This ultimately leads to improved long-term electricity production and integration with smart grid systems for optimized energy management.

The research presented here contributes to the growing body of work that investigates the application of deep learning techniques for monitoring and optimizing PV systems. By providing a novel and practical approach to quantify cooling efficiency, this study paves the way for further advancements in renewable energy technology solutions. The proposed deep regression analysis method has the potential to significantly impact the efficiency and sustainability of photovoltaic energy systems.

#### Limitations

- This research focuses on quantifying cooling efficiency, and further research is needed to explore the application of deep learning for other aspects of PV system performance, such as predicting energy output or identifying potential faults.
- The accuracy of the proposed method may be affected by factors such as image quality, lighting conditions, and the presence of obstructions. Further investigation is needed to assess the robustness of the method under different conditions.
- The current dataset is limited in size and scope, and further data collection and annotation are required to improve the model’s generalization ability and ensure its applicability to a wider range of PV systems.
- The cost of implementing the proposed system needs to be further investigated to determine if it is economically viable for smaller-scale PV installations.

#### Future work

- Expanding the dataset: Collecting a larger and more diverse dataset of thermal images from various PV systems under different conditions will significantly improve the model’s generalization ability and accuracy.
- Developing more sophisticated models: Exploring more advanced deep learning architectures, such as recurrent neural networks or attention mechanisms, to further improve the accuracy and efficiency of the cooling efficiency estimation.
- Integrating with other monitoring systems: Integrating the proposed system with other monitoring systems, such as those that track weather conditions, panel performance, and grid connectivity, to provide a more comprehensive view of the PV system’s overall health.
- Exploring applications beyond cooling efficiency: Investigating the application of deep learning techniques for other aspects of PV system performance, such as predicting energy output, identifying potential faults, and optimizing energy harvesting.
- Developing a user-friendly interface: Developing a user-friendly interface that allows operators to easily access and interpret the data generated by the proposed system.

This research provides a strong foundation for future advancements in the field of photovoltaic thermal management optimization. By addressing the limitations and pursuing the proposed future work, this research can contribute significantly to the development of more efficient and sustainable PV systems.

## Conclusions

This study has presented a novel deep-learning approach for evaluating the cooling effectiveness of photovoltaic (PV) solar panels using thermal imaging videos. By employing a U-Net architecture for image segmentation, we have successfully automated the classification of cropped panels based on their cooling efficiency. This process has resulted in the creation of a comprehensive labeled dataset of thermal images, encompassing a broad range of cooling efficiencies from 0 to 100%. Our evaluation of two predictive models, a traditional three-layer Feedforward Neural Network (FNN) and a Convolutional Neural Network (CNN), has demonstrated the CNN’s superior performance in terms of root mean squared error (RMSE), mean absolute error (MAE), and R-square value. The CNN’s RMSE of 2.3%, MAE of 1.2%, and R-square of 0.95 significantly outperform the FNN’s results, indicating a more accurate and reliable method for estimating cooling efficiencies. While these results are encouraging, we acknowledge the limitations of our research. The models were developed and validated under specific conditions, and their applicability to different types of PV panels and environmental settings remains to be fully investigated. The generalizability of our findings to real-world scenarios, where factors such as varying panel materials, designs, and unpredictable environmental influences come into play, requires further testing. To address these limitations, future work will focus on expanding the diversity of the dataset to include a wider array of PV panel types and environmental conditions. Field trials will be conducted to evaluate the robustness and practicality of our models in operational settings. Additionally, we will explore the integration of additional sensor data to refine the models’ predictive capabilities. The vision-based, contactless evaluation method we have developed offers significant adaptability and potential for integration into existing PV infrastructure. This could lead to improved maintenance practices and enhanced overall system efficiency. The curated thermal imaging datasets and deep learning architectures provided by this research represent a substantial advancement in the field of renewable energy technology.

## Data Availability

The dataset and code used in this study are public and all test data are available at this portal (https://universe.roboflow.com/kafrelsheikh-university/photovoltaic_thermal_images/dataset/2 ).
